# Special Issue “Molecular Mechanisms of Memory Formation and Modification”

**DOI:** 10.3390/ijms22084113

**Published:** 2021-04-16

**Authors:** Timothy J. Jarome, Janine L. Kwapis

**Affiliations:** 1 Department of Animal and Poultry Sciences, Virginia Polytechnic Institute and State University, Blacksburg, VA 24061, USA; 2 School of Neuroscience, Virginia Polytechnic Institute and State University, Blacksburg, VA 24061, USA; 3 Department of Biology, Pennsylvania State University, University Park, PA 16802, USA; 4 Center for the Molecular Investigation of Neurological Disorders (CMIND), Pennsylvania State University, University Park, PA 16802, USA

## 1. Introduction

Memory is vital to human functioning and controls future behavioral responses. Many neurological, neurodegenerative, and psychiatric disorders are associated with memory impairments or the presence of maladaptive memories. Thus, understanding the molecular mechanisms that control both normal and abnormal memory formation and modification is critical for treating a variety of disorders characterized by altered memory function.

This Special Issue is a compilation of 11 research papers and reviews, all covering various “Molecular Mechanisms of Memory Formation and Modification”. The topics covered in this Special Issue can be divided into two categories, (1) memory consolidation, and (2) memory reconsolidation. For memory consolidation, included in this Special Issue are three research papers and three review articles. For the former, Smith and colleagues show that proteasome-dependent degradation of the activating transcription factor 4 (ATF4), a potent inhibitor of the cAMP-response element binding protein (CREB), occurs during the induction of long-term potentiation (LTP), the proposed cellular analog of memory, via its phosphorylation by cAMP-dependent protein kinase [1]. This regulation of ATF4 may be an important mechanism for controlling gene expression changes during activity- and learning-dependent synaptic plasticity in cells. Kornhuber and Zoicas examined the temporal dynamics of the requirement for protein synthesis in the consolidation of social fear memory [2]. They found that systemic injections of the protein synthesis inhibitor anisomycin impaired long-term memory for a social fear conditioning task when administered 20 min prior to or immediately, 6 h or 8 h, but not 4 h, after behavioral training. These results suggest that two waves of de novo protein synthesis are required for the consolidation of social fear memories. Finally, Ferrara and colleagues used in vivo recordings of local field potentials (LFPs) evoked by stimulation of prefrontal cortical (PFC) or auditory thalamic (MgN) inputs to the basolateral amygdala (BLA) to test if PFC inhibition of the BLA following fear learning was greater in adults than adolescents [3]. They found that in adult rats, stimulation of MgN inputs enhanced BLA LFP and increased PFC inhibition of MgN inputs following fear conditioning, suggesting that fear conditioning increases inhibitory capacity of PFC inputs to the BLA in adults, but this enhancement is weaker in adolescents.

In addition to these research papers, three review articles were included in this Special Issue that all discussed epigenetic mechanisms of memory consolidation. Leonetti and colleagues reviewed the role of methylation of the adenosine base (m6A) of RNA in neuronal development and memory formation, emphasizing the overlap in functionality of m6A in these two processes [4]. Importantly, they discuss how m6A-mediated “translational priming” during consolidation could regulate memory locally at synapses. Creighton and colleagues reviewed the epigenetic factors involved in memory consolidation in young, healthy adults and how these mechanisms are altered with age [5]. They conclude by discussing the potential source for age-related changes in the epigenome and how targeting of this has important implications for the development of therapeutic interventions for age-associated memory loss. Finally, Lopez and colleagues review the role of histone modifying and chromatin remodeling enzymes in memory consolidation, highlighting the emerging evidence implicating ATP-dependent chromatin remodeling activity-dependent plasticity in the brain [6]. Additionally, they discuss how chromatin remodeling complexes may contribute to the development of substance use disorders.

This Special Issue also includes five articles focused on memory retrieval and reconsolidation: four research papers and one review. All of these articles focus on what happens to memory after the initial consolidation period is over and the memory is placed into long-term storage. Traditionally, memories were thought to be resistant to disruption once consolidation was complete, but more recent evidence has demonstrated that stored memories become labile again when the animal is presented with a reminder cue or retrieval event. This period of transient susceptibility and restabilization of memory, termed “reconsolidation” has generated intense interest as a potential intervention to weaken, update, or even erase maladaptive associations. First, Trask and colleagues demonstrate that both middle-aged (15-month-old) and aged (22-month-old) rats show deficits in retrieving memory for trace fear conditioning, a form of aversive learning that is particularly susceptible to the effects of aging [7]. Then, categorizing the rats as age-impaired or age-unimpaired, they demonstrate that these memory retrieval deficits are accompanied by impaired protein degradation and a corresponding accumulation of zif268 protein in multiple brain regions necessary for trace fear memory. Age-related memory impairments may therefore stem from disruptions in protein degradation that contribute to the accumulation of proteins and, ultimately, impaired memory retrieval.

The remaining articles focus on understanding the mechanisms that underlie the destabilization and restabilization of memory following retrieval. Navabpour and colleagues [8] investigated DNA double-strand breaks (DSBs) as a mechanism that may control the transcriptional program necessary for memory restabilization. They demonstrate that DSBs co-localize with an epigenetic marker of active transcription, H3 lysine-4 trimethylation (H3K4me3), consistent with reports that DSBs often occur at recently transcribed genes. The authors demonstrate that a marker of DSBs (H2A.XpS139) and H3K4me3 both increase at the *Npas4* gene (but not at *cFos*) shortly after retrieval, suggesting these two markers of transcriptional activity may function in concert to support reconsolidation. Impressively, knockdown of the enzyme responsible for DSBs (topoisomerase IIβ) before retrieval reverses these effects and impairs memory reconsolidation, demonstrating that DSB-induced transcriptional increases are critical for successful reconsolidation. Elahi and colleagues take a different approach to disrupting reconsolidation, attempting to use electroconvulsive shock (ECS) as a non-surgical intervention to interfere with reconsolidation [9]. Despite numerous historical reports that ECS can impair reconsolidation, in their experiments, the authors found no evidence that post-retrieval ECS impairs reconsolidation for either cued or context fear memory. In a clever follow-up experiment, Elahi and colleagues tested the effects of ECS delivered immediately after each of four daily extinction sessions and find that ECS does impair the consolidation of extinction memory. Overall, this work indicates that ECS is not an effective intervention for disrupting reconsolidation, underscoring a need for more targeted and effective pharmaceuticals that can disrupt the reconsolidation process in a minimally invasive manner. One potential treatment could be M_1_ muscarinic agonists, identified by Wideman and colleagues as a mechanism capable of driving memory destabilization even in the face of boundary conditions that prevent reconsolidation, like the age of the original memory [10]. In a series of experiments, the authors show a beautiful molecular dissociation in the perirhinal cortex between memory destabilization, supported by GluN2B-containing NMDA receptors (NMDARs) and its subsequent restabilization, supported by GluN2A-containing NMDARs. They then demonstrate that cholinergic activity at M_1_ muscarinic receptors can override the effects of glutamate (mediated via GluN2A- and GluN2B-containing NMDARs), with an M_1_ agonist driving destabilization even in the presence of a GluN2B inhibitor that normally blocks destabilization. The M_1_ mAChR may therefore be a novel target capable of forcing an otherwise resistant memory to become labile and susceptible to modification. Finally, Bellfy and Kwapis review the evidence that the reconsolidation process may allow existing memories to update in response to new, relevant information [11]. In their review, the authors outline the major behavioral paradigms used to study reconsolidation and describe the different molecular mechanisms across the brain that support this process of reconsolidation-dependent memory updating. Together, these articles converge on the idea that understanding the mechanisms that support memory retrieval and reconsolidation and understanding why these processes fail in conditions like old age is critically important to develop treatments to boost memory when it fails or reduce the emotional content of memory when it is maladaptive.

In conclusion, this Special Issue covered a range of molecular mechanisms that support both the consolidation and the reconsolidation of memory. Understanding how each of these phases of memory work at a mechanistic level is a key step toward treating the myriad disorders characterized by alterations in memory.

## Data Availability

Not applicable.
